# The Influence of Lumbar and Lumbosacral Segmental Fusion as a Predictor of Sacroiliac Joint Pain

**DOI:** 10.3390/jcm15072696

**Published:** 2026-04-02

**Authors:** Fouad Qupti, Mohammad Walid Al-Smadi, Árpád Viola, Kamal Haider, Jeries Hakim, Mokbil Shalah, Khaled Aslan

**Affiliations:** 1 Orthopedic Department, EMMS Nazareth Hospital, Nazareth 16100, Israel; fo2ad_16@hotmail.com (F.Q.); haiderkamal1med@gmail.com (K.H.); jeries_hakim@nazhosp.com (J.H.); mokbel@nazhosp.com (M.S.); 2 Department of Neurosurgery and Neurotraumatology, Dr. Manninger Jenő Traumatology Institution, 1081 Budapest, Hungary; smadi996@hotmail.co.uk (M.W.A.-S.); arpadviola@gmail.com (Á.V.); 3 Department of Neurotraumatology, Semmelweis University, 1081 Budapest, Hungary

**Keywords:** lumbar, lumbo-sacral segmental fusion, sacroiliac joint pain

## Abstract

**Objective:** To evaluate the influence of lumbar and lumbo-sacral spinal fusion surgery on the incidence of postoperative sacroiliac joint (SIJ) pain. **Methods:** A retrospective analysis was conducted on 154 patients who underwent lumbar or lumbo-sacral spinal fusion between 2015 and 2019 at EMMS Nazareth Hospital, Israel. Clinical data, surgical details, and demographic factors were collected. Postoperative SIJ pain was diagnosed primarily through the FABER test and confirmed by selective injections and imaging when clinically indicated. Statistical analyses included Chi-square and Student’s *t*-tests. **Results:** Postoperative SIJ pain was observed in 28.5% of patients. The incidence significantly correlated with the number of fused segments (*p* = 0.048) and the involvement of sacral segments (36.1% with sacral involvement vs. 16.7% without; *p* = 0.009). Demographic factors (age, gender, ethnicity) were not significant predictors. **Conclusions:** Lumbar and lumbo-sacral spinal fusions substantially increase the risk of postoperative SIJ pain, particularly with multi-segment and sacral-involved surgeries. Careful preoperative surgical planning is essential to minimize SIJ-related complications and improve patient outcomes.

## 1. Introduction

The sacroiliac joint (SIJ) is a key load-bearing articulation responsible for transmitting forces between the spine and pelvis, and is therefore subjected to substantial biomechanical stress during upright posture, ambulation, and load transfer [1]. Dysfunction of this joint commonly presents as localized pain in the lower lumbar region, buttocks, or posterior thigh, often mimicking lumbar spine pathology and complicating clinical evaluation [2]. Although SIJ pain may result from trauma, inflammatory disorders, pregnancy-related ligamentous laxity, or degenerative processes, an increasingly recognized contributor is lumbar and lumbosacral spinal fusion, which significantly alters spinopelvic biomechanics and increases mechanical loading on the SIJ [3].

Lumbar and lumbosacral fusion procedures have become widely utilized for treating degenerative disc disease, spondylolisthesis, lumbar stenosis, mechanical instability, and spinal deformities. These surgeries, typically employing pedicle-screw constructs and interbody cages, aim to eliminate painful segmental motion and restore stability [4]. Despite reported success rates of 70–90%, a considerable proportion of patients continue to experience persistent or recurrent pain following surgery, a constellation frequently referred to as Failed Back Surgery Syndrome (FBSS) [5]. In this context, the SIJ has emerged as a substantial but often under-recognized pain generator, with prior reports suggesting that SIJ dysfunction accounts for up to 25% of chronic low back pain after fusion [6]. As such, understanding the risk factors and mechanisms underlying post-fusion SIJ pain is critical for improving diagnostic accuracy and patient outcomes.

Biomechanical investigations have demonstrated that spinal fusion, particularly when extending across multiple lumbar segments or involving sacral fixation, restricts lumbar mobility and consequently increases stress, rotational forces, and shear loads across the SIJ [7]. Finite-element modeling studies confirm that both the magnitude and distribution of SIJ stress rise markedly after fusion, supporting the premise that altered kinematics predispose the joint to degeneration and symptomatic overload [8]. Clinically, several studies have shown that the number of fused levels and sacral involvement correlate strongly with the risk of SIJ degeneration and postoperative pain, reinforcing the concept that immobilization of lumbar motion segments shifts compensatory stress toward the SIJ [9].

Recent high-quality evidence further strengthens these associations. A 2024 systematic review by Karimi et al. synthesized current data on post-fusion SIJ dysfunction and reported that multilevel fusion, fixed fusion constructs, and lumbosacral fixation are significant predictors of new-onset SIJ pain [5]. Their review also highlighted diagnostic challenges, noting that provocation tests such as FABER possess limited specificity, and that image-guided SIJ blocks remain essential for establishing a definitive diagnosis. These findings emphasize the need for consistent and rigorous diagnostic criteria in postoperative follow-up.

Complementary evidence from Xu et al. in 2024 provides a meta-analytic perspective, reporting a pooled incidence of 9.4% for new-onset SIJ pain after spinal surgery and identifying female sex, fusion across multiple segments, fusion to the sacrum, and increased postoperative pelvic tilt as significant risk factors [3]. Their analysis demonstrated that postoperative changes in lumbar lordosis and sacral slope further contribute to SIJ overload, highlighting the interplay between sagittal alignment parameters and postoperative SIJ pain. These results align with biomechanical models suggesting that both fusion length and sacral fixation alter spinopelvic dynamics in a manner that predisposes the SIJ to symptomatic degeneration.

Further supporting these conclusions, Shin et al. (2024) reported that SIJ degeneration is significantly more common among patients undergoing fusion of three or more lumbar segments and among those whose constructs terminate at S1 [6]. Their work confirms that both surgical extent and sacral involvement play central roles in modifying SIJ stress distribution and degeneration risk. Collectively, these contemporary studies underscore that postoperative SIJ pain is not merely a secondary adjunct phenomenon but a predictable biomechanical consequence of lumbar and lumbosacral fusion.

Despite these insights, important gaps remain, particularly regarding patient-specific predictors, demographic influences, and diagnostic consistency. Variability in diagnostic criteria across studies, such as reliance on FABER alone, inconsistent use of SIJ blocks, and differences in radiographic evaluation contributes to variability in reported incidence. As emphasized by Karimi et al. and Xu et al., SIJ pain remains frequently underdiagnosed or misattributed to recurrent lumbar pathology, underscoring the need for structured postoperative evaluation algorithms [3,5,10]. Given these challenges, additional data from diverse clinical settings are needed to clarify the incidence and risk factors associated with SIJ pain following spinal fusion.

Accordingly, the present study aims to determine the incidence of new-onset SIJ pain following lumbar and lumbosacral fusion in our institution and to evaluate the influence of demographic factors, the number of fused segments, and sacral involvement on the development of postoperative SIJ pain. Understanding these relationships may help refine surgical planning, enhance patient counseling, and improve postoperative diagnostic accuracy.

## 2. Materials and Methods

### 2.1. Study Design

This retrospective observational study examined patients who underwent lumbar or lumbosacral spinal fusion at the Orthopedic Department of the English Hospital in Nazareth between January 2015 and December 2019. The retrospective design enabled a comprehensive evaluation of postoperative outcomes across a large clinical cohort, with a specific focus on the incidence and determinants of new-onset sacroiliac joint (SIJ) pain after spinal fusion. All procedures were performed by a single senior orthopedic spine surgeon to ensure consistency in surgical technique, instrumentation, and perioperative management, thereby reducing variations that might otherwise influence postoperative SIJ-related symptoms.

### 2.2. Participants and Data Collection

A total of 192 patients were initially identified through the institution’s electronic health record system (Prometheus). Inclusion criteria encompassed all adult patients, regardless of age or gender, who underwent instrumented lumbar or lumbosacral fusion during the study period. To maintain methodological rigor, exclusion criteria were applied to patients with the following conditions: (1) a documented history of chronic SIJ pain prior to surgery; (2) confirmed rheumatologic or inflammatory joint disease; (3) spinal fractures requiring fusion; and (4) failure to attend postoperative follow-up evaluations. After applying these exclusions, 154 patients remained eligible for final analysis.

Given the known biomechanical significance of sacral involvement, the cohort was stratified into two groups based on fusion anatomy:

1. patients whose fusion constructs extended to include S1, and
2. patients whose fusion terminated above the sacrum.

This stratification allowed for targeted analysis of the impact of sacral fixation on postoperative SIJ pain.

### 2.3. Diagnostic Evaluation

Postoperative SIJ pain was assessed using a structured, multi-step diagnostic approach. As part of routine clinical follow-up, all patients underwent physical examination that included the FABER (Flexion, Abduction, External Rotation) test, commonly utilized to provoke SIJ-related symptoms. The FABER test was performed with the patient in the supine position; reproduction of pain localized to the SIJ region was recorded as a positive finding. Because this assessment formed part of standard postoperative care, no additional consent was required.

Recognizing the limited specificity of provocation tests, patients presenting with persistent buttock or lower lumbar pain suspicious for SIJ origin were further evaluated using confirmatory diagnostic modalities. When clinically indicated, patients underwent image-guided SIJ injections utilizing local anesthetic. A reduction of at least 50 percent in reported pain following injection was considered diagnostic for SIJ-origin pain. In addition, selective imaging modalities, including plain radiographs, computed tomography (CT), or magnetic resonance imaging (MRI) were employed to exclude alternative pain generators and support diagnostic accuracy. This combined approach aimed to improve reliability and minimize diagnostic misclassification, a known limitation in purely clinical SIJ assessment.

### 2.4. Follow-Up and Data Acquisition

All patients were monitored for a minimum of two years following surgery as part of standard postoperative care. Follow-up evaluations occurred at regular clinic intervals, during which clinical symptoms, physical examination findings, and imaging results were documented. Demographic data including age, gender, and ethnicity were systematically collected to explore potential associations with postoperative SIJ pain. Surgical variables including the number of fused vertebral segments, presence or absence of sacral involvement, and specific fusion constructs were extracted from operative reports and radiographic documentation.

The primary study outcome was the development of new-onset SIJ pain within the two-year postoperative period. Secondary variables included demographic factors and surgical characteristics potentially associated with SIJ pain.

### 2.5. Statistical Analysis

Statistical analyses were conducted using SPSS software version 20 (IBM Corp., Armonk, NY, USA) and Microsoft Excel (Version 2603, Build 19822.20104). Descriptive statistics were generated for demographic, surgical, and clinical variables. Categorical variables such as presence of postoperative sacroiliac joint (SIJ) pain, gender, ethnicity, and sacral involvement were compared using Pearson’s chi-square test. Continuous variables, including age, were analyzed using Student’s *t*-test.

In addition, multivariate logistic regression analysis was performed to identify independent predictors of postoperative SIJ pain, including age, sex, number of fused segments, and sacral involvement. Odds ratios (ORs) with 95% confidence intervals (CIs) were calculated. Statistical significance was defined as *p* < 0.05.

### 2.6. Ethical Considerations

The study was approved by the Institutional Ethics Committee of EMMS Nazareth Hospital (NA212K). In accordance with national and institutional guidelines for retrospective studies utilizing anonymized clinical data, informed consent was waived. All data were handled confidentially and stored securely, with access restricted to authorized research personnel only.

## 3. Results

### 3.1. Demographic and Clinical Characteristics

During the study period (2015–2019), a total of 192 patients underwent lumbar or lumbosacral fusion at the Orthopedic Department of the English Hospital, Nazareth. After applying predefined exclusion criteria, including pre-existing SIJ pain (n = 2), rheumatologic disease such as fibromyalgia (n = 11), and loss to follow-up (n = 25), 154 patients qualified for final analysis. Among them, 88 (57.1 percent) were female and 66 (42.9 percent) were male. The mean age was 49.3 ± 1.3 years (range 21–78). Most participants were Arab (89.6 percent), with Jewish patients representing 10.4 percent of the cohort.

To improve clarity and reduce redundancy, demographic variables were consolidated into a single, unified Table 1.

### 3.2. Incidence of Postoperative SIJ Pain

The primary endpoint was the presence of new-onset SIJ pain within two years following spinal fusion. Out of the 154 eligible patients, 44 (28.5 percent) developed postoperative SIJ pain, while 110 (71.5 percent) remained asymptomatic. This incidence reflects a clinically meaningful proportion of patients experiencing SIJ-related symptoms after fusion.

### 3.3. Impact of Demographic Factors

#### 3.3.1. Gender

Postoperative SIJ pain occurred in 27 females (61 percent) and 17 males (39 percent). However, Chi-square testing demonstrated no statistically significant association between gender and postoperative SIJ pain (χ^2^ = 0.448, *p* = 0.503). These findings align with recent systematic reviews showing inconsistent associations between sex and SIJ dysfunction following fusion.

#### 3.3.2. Ethnicity

Similarly, ethnicity did not demonstrate a significant relationship with SIJ pain. Of those reporting pain, 95 percent were Arab and 5 percent Jewish, mirroring the overall ethnic distribution. Chi-square testing did not reveal significant differences between groups (χ^2^ = 2.260, *p* = 0.133). Although ethnicity was not a significant variable, the predominance of Arab patients may limit generalizability, a point later addressed in the Section 5.

#### 3.3.3. Age

Patients who developed SIJ pain had a slightly higher mean age (51.8 years) compared to those without pain (48.2 years), but this difference did not reach statistical significance (*p* = 0.079). When age was stratified (<65 vs. ≥65 years), differences remained non-significant (χ^2^ = 1.42, *p* = 0.234). Thus, age was not identified as a meaningful predictor.

To further evaluate independent predictors of postoperative SIJ pain, a multivariate logistic regression analysis was performed including age, sex, number of fused segments, and sacral involvement. The analysis demonstrated that sacral involvement (OR = 2.71, 95% CI 1.18–6.20, *p* = 0.018) and number of fused segments (OR = 1.45, 95% CI 1.03–2.03, *p* = 0.031) remained independently associated with postoperative SIJ pain. 

#### 3.3.4. Effect of Number of Fused Segments

**Figure 1:**
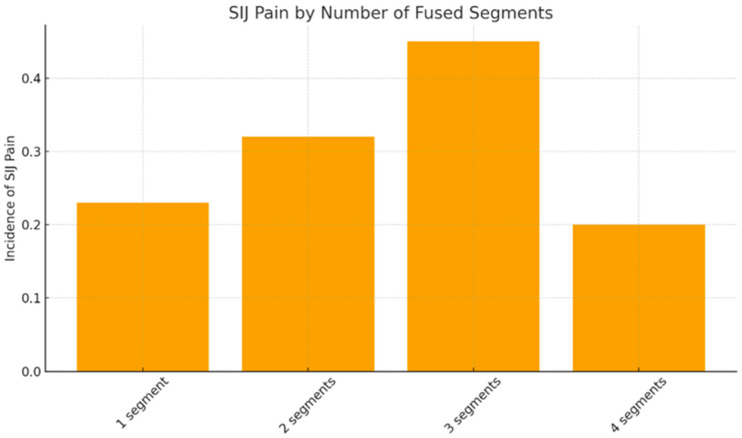
Incidence of SIJ pain by number of fused segments.

A significant association was observed between the number of fused segments (Figure 1) and postoperative SIJ pain (χ^2^ = 7.91, *p* = 0.048). Pain incidence increased progressively with each additional fused level:

1. 1-level fusion: 23% developed SIJ pain;
2. 2-level fusion: 32%;
3. 3-level fusion: 45%;
4. 4-level fusion: 20% (sample small; interpret cautiously).

These findings demonstrate a dose–response trend, consistent with contemporary biomechanical evidence showing greater SIJ loading in multilevel constructs.

#### 3.3.5. Effect of Sacral Involvement

**Figure 2:**
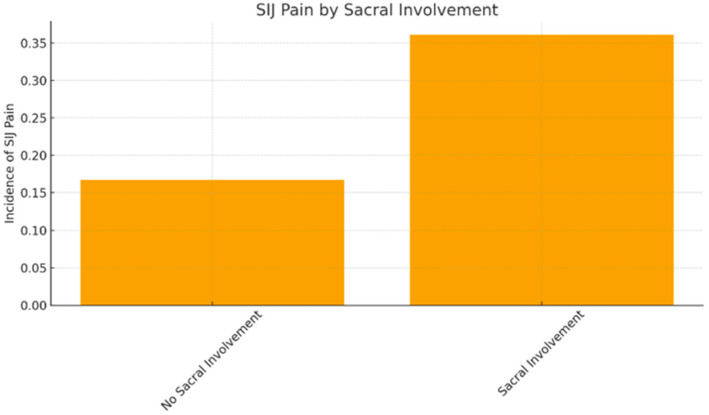
SIJ pain incidence by sacral involvement.

Sacral involvement emerged as a strong predictor of SIJ pain. Among the 94 patients whose fusion extended to S1, 36.1 percent developed SIJ pain (Figure 2). In contrast, only 16.7 percent of the 60 patients with lumbar-only fusion reported SIJ pain. This difference was statistically significant (χ^2^ = 6.826, *p* = 0.009), supporting existing literature that sacral fixation alters spinopelvic biomechanics and increases SIJ stress.

### 3.4. Detailed Analysis of Fusion Levels and SIJ Pain

Beyond the broad association with fusion length, a more granular review was conducted to evaluate the impact of specific fusion patterns on postoperative SIJ pain. The most common fusion pattern in the cohort was L4–L5–S1, representing a typical lumbosacral construct. This subgroup demonstrated the highest incidence of SIJ pain (≈42 percent). These findings are consistent with contemporary biomechanical studies showing that constructs terminating at S1 create substantial rotational and shear forces transmitted through the sacroiliac joint.

In contrast, lumbar-only constructs, particularly L3–L4, L2–L3–L4, and L2–L5 showed lower SIJ pain incidences ranging from 15 to 25 percent. These levels avoid direct sacral fixation and therefore exert less spinopelvic load redistribution. However, subgroup interpretations should remain cautious due to variability in sample size and distribution. To improve clarity, fusion levels were consolidated into Table 2.

### 3.5. Relationship Between Diagnostic Modalities and SIJ Pain Identification

Because SIJ pain diagnosis initially relied on the FABER test, additional stratification was performed to examine the proportion of patients undergoing confirmatory diagnostic procedures. Out of the 44 patients with suspected SIJ pain, 22 (50 percent) underwent image-guided SIJ injections. Among these, 18 patients (81.8 percent) reported pain relief ≥50 percent, meeting the diagnostic threshold for SIJ-origin pain.

This finding underscores two important points:

1. The FABER test likely overestimates SIJ pain when used as a standalone tool, consistent with literature showing limited specificity.
2. Combined diagnostic approaches (clinical + injection) provide higher diagnostic confidence and align with current international recommendations.

However, the fact that only half of the symptomatic patients received confirmatory injections highlights a limitation in the diagnostic consistency—a point later emphasized in the Limitations section.

Chi-square tests:

- Pearson χ^2^ = 6.826, df = 1, *p* = 0.009;
- Likelihood Ratio = 7.174, *p* = 0.007;
- Fisher’s Exact Test (2-sided) = 0.010.

Table 3 presents a clear and statistically significant association between sacral involvement and postoperative sacroiliac joint (SIJ) pain. Among patients whose fusion constructs extended to S1, 34 out of 94 individuals (36.1%) developed SIJ pain compared with only 10 out of 60 patients (16.7%) whose fusion was limited to lumbar levels. This nearly twofold increase demonstrates that fusion involving the sacrum imposes substantially greater biomechanical stress on the SIJ. The statistical validity of this association was confirmed by multiple tests: the Pearson chi-square value of 6.826 (*p* = 0.009) indicates significance, as do the likelihood ratio (*p* = 0.007) and Fisher’s Exact Test (*p* = 0.010). The absence of cells with expected counts below five ensures that the chi-square assumptions were met, strengthening the robustness of the analysis. Clinically, this reinforces the concept that sacral fixation transforms the lumbopelvic mechanics by eliminating motion at the L5–S1 junction, thereby shifting compensatory forces to the SIJ. These findings align with contemporary literature identifying sacral involvement as a major risk factor for postoperative SIJ degeneration and pain.

Table 4 therefore highlights the clinical importance of carefully evaluating the need for sacral anchoring during surgical planning and presents the results of the multivariate logistic regression analysis assessing independent predictors of postoperative SIJ pain. Sacral involvement (OR = 2.71, 95% CI 1.18–6.20, *p* = 0.018) and the number of fused segments (OR = 1.45, 95% CI 1.03–2.03, *p* = 0.031) were identified as significant predictors. In contrast, age (OR = 1.02, *p* = 0.21) and sex (OR = 1.18, *p* = 0.64) were not statistically significant predictors. The detailed results are presented in Table 4.

### 3.6. Comparison of Imaging Findings

Relevant imaging, including pelvic X-rays, CT, and MRI, was reviewed selectively in patients with persistent symptoms. Radiological SIJ degeneration patterns included joint space narrowing, subchondral sclerosis, and osteophyte formation. Among the 22 patients who underwent imaging evaluation:

1. 59 percent demonstrated radiographic evidence consistent with SIJ degenerative change;
2. 41 percent showed normal radiographic SIJ anatomy despite pain symptoms.

These findings emphasize the known disconnect between SIJ imaging and clinical presentation; radiographic findings alone are insufficient for diagnosis and must be interpreted within a clinical context.

## 4. Discussion

The present study demonstrates that postoperative sacroiliac joint (SIJ) pain is a clinically significant complication following lumbar and lumbosacral fusion, with an incidence of 28.5 percent in our cohort. This rate is higher than those reported in many prior studies, in which incidence typically ranged from 7 to 25 percent [1,2]. Xu et al. reported a pooled rate of 9.4 percent in a 2024 meta-analysis, suggesting that differences in patient demographics, fusion extent, diagnostic criteria, and follow-up duration may explain the variability between studies [3]. Our higher rate may reflect a greater proportion of multi-segment and lumbosacral constructs, both recognized contributors to SIJ dysfunction [4,5,6].

A key finding is the strong association between the number of fused segments and postoperative SIJ pain. We observed a clear dose–response pattern, with SIJ pain rising from 23 percent in single-level fusions to 45 percent in three-level fusions. These results closely mirror the findings of Unoki et al. and other researchers who demonstrated that multi-segment fusion substantially increases SIJ loading and degeneration [7,8]. This relationship is further reinforced by the 2024 systematic review by Karimi et al., which identified multi-level and fixed constructs as leading predictors of SIJ dysfunction [5]. The convergence of our findings with contemporary literature highlights fusion length as one of the most robust determinants of postoperative SIJ pain [6,9].

Another major predictor in our cohort was sacral involvement, with 36.1 percent of S1 fusions resulting in SIJ pain compared to only 16.7 percent of lumbar-only fusions [5,6]. This substantial increase supports extensive biomechanical evidence demonstrating that anchoring spinal constructs into the sacrum dramatically alters spinopelvic force distribution. Finite-element studies consistently showed greater angular motion, shear stress, and ligament loading across the SIJ following lumbosacral fusion [2,11]. Clinically, these findings align with the comparative study by Shin et al. (2024), which identified sacral fixation as a key driver of radiographic SIJ degeneration. Our findings reinforce the interpretation that sacral involvement independently contributes to postoperative SIJ pathology [5,6].

The multivariate logistic regression analysis confirmed that both the number of fused segments and sacral involvement were independent predictors of postoperative SIJ pain. Specifically, sacral involvement had an OR of 2.71 (95% CI 1.18–6.20, *p* = 0.018), and the number of fused segments had an OR of 1.45 (95% CI 1.03–2.03, *p* = 0.031), while age and sex were not significantly associated [5,6].

Demographic variables including age, gender, and ethnicity were not significantly associated with SIJ pain in our population [3,7]. Although Xu et al. identified female sex as a potential risk factor in their meta-analysis, our results did not reproduce this finding [3]. This discrepancy may be due to differences in cultural pain reporting, sample distribution, or underlying population heterogeneity. The lack of significance for age also contrasts with other studies reporting age-associated susceptibility to degenerative changes; however, these associations are typically modest and inconsistent across cohorts [7,12]. Importantly, the demographic composition of our study (nearly 90 percent Arab) limits external generalizability and reduces statistical power to detect ethnic differences [7,12,13].

Fusion patterns also influenced postoperative SIJ pain. The L4–L5–S1 construct demonstrated the highest incidence (≈42 percent), consistent with studies showing that constructs involving S1 impose greater rotational and translational forces on the SIJ [1,5,6]. Earlier work by Colo et al. and others showed significant increases in SIJ degeneration and low back pain following fusions that terminate at S1 [1,4,6]. This aligns with our observation that L4–L5–S1 fixation represents a high-risk pattern, underscoring the profound biomechanical consequences of sacral anchoring [1,5,6].

The biomechanical foundation for our findings is strongly supported by the existing literature. Fusion of lumbar segments, particularly when extending to the sacrum, fundamentally alters load transfer across the pelvis. Finite-element studies have demonstrated increases in SIJ angular motion, sacral slope changes, and compensatory ligament tension after fusion [2,11]. Ivanov et al. showed that immobilizing lumbar segments significantly increases stress across the SIJ, while Zhao et al. confirmed increased shear forces in multi-segment and lumbosacral constructs [11]. Our study provides clinical validation for these biomechanical models, demonstrating that both sacral involvement and fusion length correlate directly with SIJ pain.

Diagnostic considerations also influence reported incidence. In our cohort, the FABER test was used universally as the initial screening tool [9]. Although FABER is widely used, it is well-known to have limited specificity. Only 50 percent of patients with suspected SIJ pain underwent confirmatory image-guided injections, yet more than 80 percent of those showed ≥50 percent pain relief after anesthetic block [9]. This finding reinforces the diagnostic recommendation emphasized by Karimi et al. and Xu et al., who noted that SIJ blocks remain the gold standard for confirming SIJ-origin pain [3,5]. Variability in the use of confirmatory injections across studies likely contributes to the wide range of SIJ incidence reported in the literature [4,5,6].

The discrepancy between our incidence rate (28.5 percent) and lower pooled estimates from large meta-analyses may also reflect differences in baseline spinopelvic parameters, which were not evaluated in this study [3]. Xu et al. demonstrated that postoperative pelvic tilt is an important predictor of SIJ pain, suggesting that sagittal alignment changes play a crucial role in symptom development [3]. Because our dataset lacked consistent radiographic evaluation of pelvic parameters, we were unable to assess their influence. This limitation highlights the need for standardized spinopelvic imaging in future research [3]

Our demographic findings also provide additional context. Age, gender, and ethnicity were not significant predictors of SIJ pain in this cohort. Although some studies have proposed age-related susceptibility to degeneration or sex-related differences in pelvic anatomy, our results align more closely with studies demonstrating minimal demographic impact. The lack of association in our cohort may reflect the dominance of biomechanical factors over biological variability. However, because our population was highly homogeneous (≈90 percent Arab), the ability to detect ethnic differences was limited. Future studies with more diverse populations may provide further clarity [2,9,12].

The clinical implications of our findings are highly relevant to surgical planning and postoperative management. First, surgeons should recognize that multi-level fusion and S1 fixation substantially increase the risk of SIJ pain [5,6,10]. This information should be communicated clearly during preoperative counseling, particularly for patients undergoing fusion beyond two levels or involving the sacrum. Second, when clinically feasible, limiting fusion length may help reduce postoperative SIJ complications [5,6]. Third, preoperative screening for SIJ dysfunction should be considered in individuals with lumbosacral pathology, as pre-existing SIJ abnormalities may worsen after fusion. Lastly, in patients presenting with persistent buttock or low lumbar pain after fusion, early consideration of SIJ-origin pain can improve diagnostic efficiency and guide targeted therapy [9,10]. These recommendations are supported by evidence from Karimi et al., Shin et al., and Unoki et al., who collectively emphasize the biomechanical and clinical importance of the SIJ in post-fusion outcomes [5,6,10].

Despite the valuable contributions of this study, limitations must be acknowledged. As a retrospective, single-center analysis, the findings may not be generalizable to the broader population [5,6,10]. Diagnostic variability, particularly the reliance on provocation tests without universal confirmatory injections, may have contributed to the under- or over-estimation of SIJ pain incidence [9]. Additionally, the lack of spinopelvic radiographic parameters and the demographic homogeneity of the cohort limit the ability to fully contextualize risk [3,7]. These limitations align with constraints noted in prior studies, emphasizing the need for prospective, multi-center research with standardized diagnostic protocols [5,6,10].

In summary, this study reinforces the critical role of biomechanical factors in postoperative SIJ pain and demonstrates strong alignment with contemporary evidence from 2024 and earlier foundational work. Our results confirm that both fusion length and sacral involvement are key predictors of SIJ dysfunction, highlighting the need for refined surgical planning and improved diagnostic evaluation to mitigate this common postoperative complication [11,13].

## 5. Limitations

This study has several limitations that should be acknowledged when interpreting the findings. First, the retrospective, single-center design introduces the potential for selection and reporting bias, which may limit the generalizability of the results. Second, although all patients were screened clinically using the FABER test, only a subset of symptomatic individuals underwent image-guided SIJ injections, which represent the diagnostic gold standard; therefore, some degree of diagnostic misclassification cannot be excluded. Third, radiographic spinopelvic parameters such as pelvic tilt, sacral slope, and lumbar lordosis were not uniformly collected, preventing assessment of their influence despite evidence from recent studies indicating their relevance to SIJ dysfunction. Fourth, the study population was demographically homogeneous, with nearly 90 percent of patients identifying as Arab, limiting the ability to examine ethnic or cultural variability in postoperative SIJ pain. Fifth, although multivariate logistic regression analysis was performed to identify independent predictors of postoperative SIJ pain, the number of variables included in the model was limited due to the sample size and number of events.

Finally, subgroup analyses of specific fusion patterns were limited by small sample sizes, requiring cautious interpretation and validation in larger, prospective, multi-center cohorts.

The present study contributes important clinical and biomechanical insights by demonstrating that both multilevel fusion and sacral involvement significantly increase the risk of postoperative sacroiliac joint (SIJ) pain. These findings reinforce the need for careful preoperative planning and highlight the SIJ as a critical adjacent structure affected by spinal fusion. Clinically, the study supports incorporating SIJ screening and targeted diagnostic blocks into postoperative evaluation algorithms. Future research should include prospective, multi-center studies with standardized imaging protocols, assessment of spinopelvic parameters, and multivariate modeling to better define high-risk patient profiles and to develop strategies that reduce fusion-related SIJ complications.

## Figures and Tables

**Table 1:** Demographic characteristics of the study population by SIJ pain status.

Variable	No SIJ Pain (n = 110)	SIJ Pain (n = 44)	Total (n = 154)
Mean age (years)	48.2 ± 1.2	51.8 ± 1.4	49.3 ± 1.3
Male (%)	49 (45%)	17 (39%)	66 (42.9%)
Female (%)	61 (55%)	27 (61%)	88 (57.1%)
Arab (%)	96 (87.3%)	42 (95%)	138 (89.6%)
Jewish (%)	14 (12.7%)	2 (5%)	16 (10.4%)

**Table 2:** SIJ pain incidence by specific fusion patterns.

Fusion Pattern	Total Patients	SIJ Pain (%)
L4–L5–S1	36	42%
L3–L4–L5	24	25%
L2–L3–L4	18	22%
L2–L5	12	17%
L5–S1 only	28	32%
Other combinations	36	19%

Note: Rates for patterns with fewer than 10 cases were excluded due to statistical underpowering.

**Table 3:** Association between sacral involvement and postoperative SIJ pain.

Sacral Involvement	No SIJ Pain (n = 110)	SIJ Pain (n = 44)	Total (n = 154)	SIJ Pain (%)
No (Lumbar-only fusion)	50	10	60	16.7%
Yes (Fusion including S1)	60	34	94	36.1%
Total	110	44	154	28.6%

**Table 4:** Multivariate logistic regression analysis of predictors of postoperative SIJ pain.

Variable	Odds Ratio (OR)	95% CI	*p*-Value
Age	1.02	0.98–1.05	0.21
Female sex	1.18	0.58–2.39	0.64
Number of fused segments	1.45	1.03–2.03	0.031
Sacral involvement	2.71	1.18–6.20	0.018

## Data Availability

All data generated or analyzed during this study are included in this published article.
